# Electrospun Nanofibers of Polyvinylidene Fluoride Enriched with Active Antimicrobial Tannic Acid for the Improvement of the Shelf Life of Cherry Tomatoes

**DOI:** 10.3390/ma18133112

**Published:** 2025-07-01

**Authors:** Rajaram Rajamohan, Ajmal P. Muhammed, Chaitany Jayprakash Raorane, Subramaniyan Ramasundaram, Iruthayapandi Selestin Raja, Sivakumar Allur Subramanian, Seong Cheol Kim, Tae Hwan Oh, Seho Sun

**Affiliations:** 1 School of Chemical Engineering, Yeungnam University, Gyeongsan 38541, Republic of Korea; 2 Institute of Nano-Bio Convergence, Pusan National University, Busan 46241, Republic of Korea; 3 Department of Orthopedic Surgery, Dongtan Sacred Heart Hospital, College of Medicine, Hallym University, Hwaseong 18450, Republic of Korea; 4 Department of Orthopedics & Rehabilitation, Carver College of Medicine, University of Iowa, Iowa City, IA 52242, USA

**Keywords:** tannic acid, nanofibers, antifungal activity, biocompatibility, shelf-life improvement

## Abstract

Active packaging films have been an essential component in food material research to ensure the safe and efficient preservation of food, fruit, and vegetables. The shelf life of fruits and vegetables may likely be extended by covering them with high-performance nanofiber (NF) films. The selection of materials for active packaging film has been a critical factor in preventing food materials from environmental contaminants (microbes) and extending the shelf life. This study aims to develop NF-based materials for cherry tomatoes to prevent fungal and bacterial damage. Bioactive NFs were produced through an electrospinning process using tannic acid (TA) within a polyvinylidene fluoride (PVDF) template. These NFs offer a sustainable alternative to synthetic packaging for food preservation. TA was incorporated into the PVDF matrix at varying concentrations (0.4 to 1.2%). Key parameters, including moisture content, thickness, opacity, water-contact angle, and thermal shrinkage, were assessed. The physicochemical results indicate that the TA NFs are suitable for further shelf-life performance evaluations. The antifungal and antibiofilm activity of the NFs was tested, showing that the TA_1.2_ in the PVDF matrix was more effective than other concentrations. Shelf-life tests demonstrated that cherry tomatoes covered with TA_1.2_ NFs showed no surface changes for up to 4 days. Importantly, the NFs were confirmed to be non-toxic to normal cells, as evidenced by tests on mouse 3T3-L1 fibroblast cells. In summary, we have developed bioactive NFs composed of TA in a PVDF matrix that enhance the shelf life of cherry tomatoes by preventing bacterial and fungal attacks on the fruit surfaces.

## 1. Introduction

Food spoilage can result from various factors. Ethylene emission from fruits and vegetables accelerates ripening; meat undergoes oxidation, affecting color and flavor; and temperature-sensitive items like sausages spoil faster under heat. Sticky foods such as honey may adhere to packaging, leading to product loss [1]. To address these issues, food packaging has evolved into two main types: active packaging, which delays spoilage, and intelligent packaging, which monitors and communicates food quality during storage and transport [2,3,4]. A key advancement in active packaging was the incorporation of bioactive agents, notably antibacterial and antioxidant compounds, that enhance food safety and quality. These agents inhibit microbial growth and oxidative reactions, such as protein and lipid oxidation, which degrade sensory properties and nutritional value [1,2]. Additionally, moisture- and oxygen-absorbing agents improve barrier performance, further preserving food during storage.

Traditional methods such as solvent casting, thermoforming, extrusion, and compression molding have been widely used to produce conventional food-packaging films [5]. In some cases, these techniques have been adapted to incorporate nanoscale additives, leading to the development of nanocomposite packaging materials. However, these methods do not directly produce nanostructured features like continuous fibers. In contrast, electrospinning has been an emerging technique that enables the fabrication of continuous polymer nanofibers (NFs) with diverse morphologies and structures, forming fibrous films on a collector [6]. Electrospun NFs have been developed from various organic polymers, including thermoplastics (e.g., polypropylene and polystyrene), biopolymers (e.g., proteins and polysaccharides), and biocompatible polymers. Unlike conventional petroleum-based plastics, these materials can be derived from renewable, biodegradable sources [7].

NFs have shown significant promise in food packaging due to their ability to enhance mechanical strength, barrier performance, and optical properties. Electrospun NFs can also regulate the controlled release of functional agents, making them responsive to environmental changes [7,8]. Incorporating additives into these fibers enables better control over food preservation and quality. Biopolymer-based NFs improve biosafety and bioavailability, offering advantages such as high porosity, low density, breathability, and a large surface area [9]. These features make NF composites suitable for broader applications across industries like textiles, healthcare, and energy storage. Electrospun NFs derived from proteins and carbohydrates exhibit precise fiber spacing, structural stability, and biocompatibility [10]. Their functional groups enable strong interactions with bioactive ingredients, allowing for controlled release in the stomach. Additionally, they protect food from oxidation, moisture, and light, while also masking odors or flavors, immobilizing enzymes and probiotics, and enabling the creation of functional foods [11]. To meet growing demands in food production and security, integrating nanotechnology into food systems was essential for driving innovation and efficiency [12].

The present study was focused on the development of biocompatible and antimicrobial packaging materials in the form of NFs fabricated within a PVDF matrix. In this approach, TA, a naturally occurring polyphenol known for its potent antimicrobial and antioxidant properties, was incorporated as the functional agent within the PVDF NF structure. Numerous studies have previously demonstrated the antimicrobial efficacy of TA against different microbial strains [13,14,15,16,17,18,19,20]. Compared to commonly used active packaging materials such as silver nanoparticles and essential oils, TA offers advantages in terms of cost, environmental safety, and ease of incorporation. TA is inexpensive, plant-derived, and biodegradable, whereas silver nanoparticles are costly and raise concerns about toxicity. Essential oils, though natural, are volatile and require encapsulation for stability, increasing production complexity. Moreover, the electrospinning process used here was scalable and allowed uniform TA distribution, supporting its industrial potential for active food-packaging applications. In our recent investigation, we successfully reported the antibacterial activity of TA when embedded in NFs using the PVDF template [21]. The results showed a significant antibacterial effect, which was strongly dependent on the concentration of TA incorporated into the NFs. Building upon the established antimicrobial (both antibacterial and antifungal properties) of TA, we aimed to explore its potential application in food packaging, specifically for the preservation of fresh fruits. Such packaging materials can help in extending the shelf life of perishable items by inhibiting microbial growth on the surface [22]. Despite the growing interest in bio-based and functional packaging systems, to the best of our knowledge, no direct research has yet been published that combines TA-loaded PVDF NFs specifically for fruit-packaging applications. This study, therefore, addresses a novel and unexplored area in the development of advanced food-packaging materials.

## 2. Materials and Methods

### 2.1. Materials

High-purity chemical reagents used in this study were sourced from reputable suppliers to ensure the consistency and reliability of the experimental outcomes. Pure polyvinylidene fluoride (PVDF) was procured from the Korean branch of Sigma-Aldrich (Seoul, Republic of Korea). The PVDF sample had an average molecular weight of approximately 534,000 g/mol and a molecular formula of (CH_2_CF_2_)ₙ, with a reported purity of 98.1%, making it suitable for high-performance material applications such as electrospinning. Tannic acid (TA), also obtained from Sigma-Aldrich Korea, was used as a functional additive in composite preparation. The TA sample had a molecular weight of 1701.20 g/mol and a molecular formula of C_76_H_52_O_46_, with a purity of 98.9%. Its high purity ensured that no extraneous compounds interfered with the fiber formation or characterization processes. Dimethyl formamide (DMF) and distilled water were used as solvents throughout the study, particularly in the preparation of electrospinning solutions and composite mixtures. DMSO, known for its excellent solvation capabilities and compatibility with PVDF, was supplied by E-Merck Ltd. (Seoul, Republic of Korea), a recognized Korean chemical manufacturer. The consistent quality and purity of all reagents played a critical role in achieving reproducible and high-quality results in the fabrication and evaluation of the electrospun NFs materials.

### 2.2. Preparation of Solutions and the Process of Electrospinning

TA was dissolved in 10 mL of distilled water to prepare aqueous solutions with varying concentrations. Specifically, 0.401, 0.801, and 1.199 g of TA were individually weighed and dissolved to produce four different TA solutions, referred to as TA_0.4_, TA_0.8_, and TA_1.2_, respectively. To prepare the composite solutions, PVDF was added to each of the TA solutions. A total of 1.812 g of PVDF was dissolved in DMF to form a homogeneous PVDF solution, and it was named TA_0_. This PVDF solution was then added to the previously prepared TA solutions. The resulting mixtures were stirred continuously for 12 h at room temperature to ensure thorough mixing and complete dissolution of the components, allowing for the uniform distribution of TA within the PVDF matrix. The three resulting solutions TA_0.4_, TA_0.8_, and TA_1.2_ were prepared for subsequent electrospinning to produce NFs. For comparison purposes, a pure PVDF solution, without the addition of TA, was also prepared as a reference. This PVDF solution was processed under the same conditions as the composite solutions. These solutions served as the starting materials for the electrospinning process, which aimed to fabricate NFs with varying concentrations of tannic acid in the PVDF matrix.

These solutions were then dispensed using a 5.0 mL syringe fitted with a 0.60 mm metallic needle. To ensure consistent and controlled delivery of the solution during the electrospinning process, a syringe pump was employed, providing a steady flow rate of 1.0 mL/h. A high voltage of 15.0 kV was applied to the needle, which facilitated the electrospinning of the solution into NFs. The electrospun NFs were collected on aluminum foil, which was placed on a metal receiver positioned at a distance of 10 to 15 cm from the syringe needle. The electrospinning setup was maintained in a controlled environment, with the process conducted at 25 °C and 18% relative humidity to ensure optimal fiber formation. This controlled atmospheric condition was critical for achieving uniformity in fiber diameter and minimizing defects during electrospinning. After the electrospinning process, the NFs were allowed to dry in a hot-air oven overnight at 140 °C. This drying step was essential to remove any residual solvents or contaminants from the NFs, ensuring that the final product was free from unwanted chemical traces. The resulting NFs were categorized as TA_0_, TA_0.4_, TA_0.8_, and TA_1.2_, depending on the concentration of TA in the PVDF matrix. Scheme S1 provides a detailed illustration of the electrospinning process, highlighting the setup and key steps involved in the fabrication of the NFs.

### 2.3. Characterization Techniques

The electrospun NFs were thoroughly characterized using analytical techniques, including Field Emission Scanning Electron Microscopy (FE-SEM) and Differential Scanning Calorimetry (DSC). All relevant instrumental parameters, including the specific conditions and settings for both FE-SEM and DSC analysis, are provided in the Supplementary Materials for reference. 

### 2.4. Thermal Shrinkage

For thermal shrinkage analysis, square NF samples (2 × 2 cm) were heated at various temperatures (25, 50, 75, and 100 °C) for 1 h. Dimensional changes before and after heating were recorded to assess thermal stability [23,24].

### 2.5. Water-Contact Angle

To study the wettability behavior, the water-contact angles of TA NFs were determined using a contact angle analyzer (OCA 20, DataPhysics, Filderstadt, Germany). 

### 2.6. Packaging Application

The practical usability of the prepared composite films in packaging applications was investigated by examining their impact on the shelf life of fresh-cut cherry tomatoes. Fully ripened tomatoes were sourced from a local market in Gyeongsan, Republic of Korea. The tomatoes were sliced and stored at 25 °C under four different conditions: (1) without packaging (control), (2) wrapped in LDPE film, (3) wrapped in TA_0_ NFs, and (4) wrapped in TA_1.2_ NFs. The samples were monitored every day, and visual appearance and weight loss were noted to analyze the preservative ability of every packaging system.

### 2.7. Statistical Analysis

All experiments were repeated at least three times (*n* = 3), and the results were represented as Mean ± Standard deviations. One-way analysis of variance (ANOVA) was used for statistical evaluation, followed by Duncan’s multiple range test (DMRT). A *p*-Value of ≤0.05 was considered statistically significant.

## 3. Results and Discussion

### 3.1. Surface Analysis of NFs

#### 3.1.1. Analysis of Surface Morphology by SEM Image

**Figure 1:**
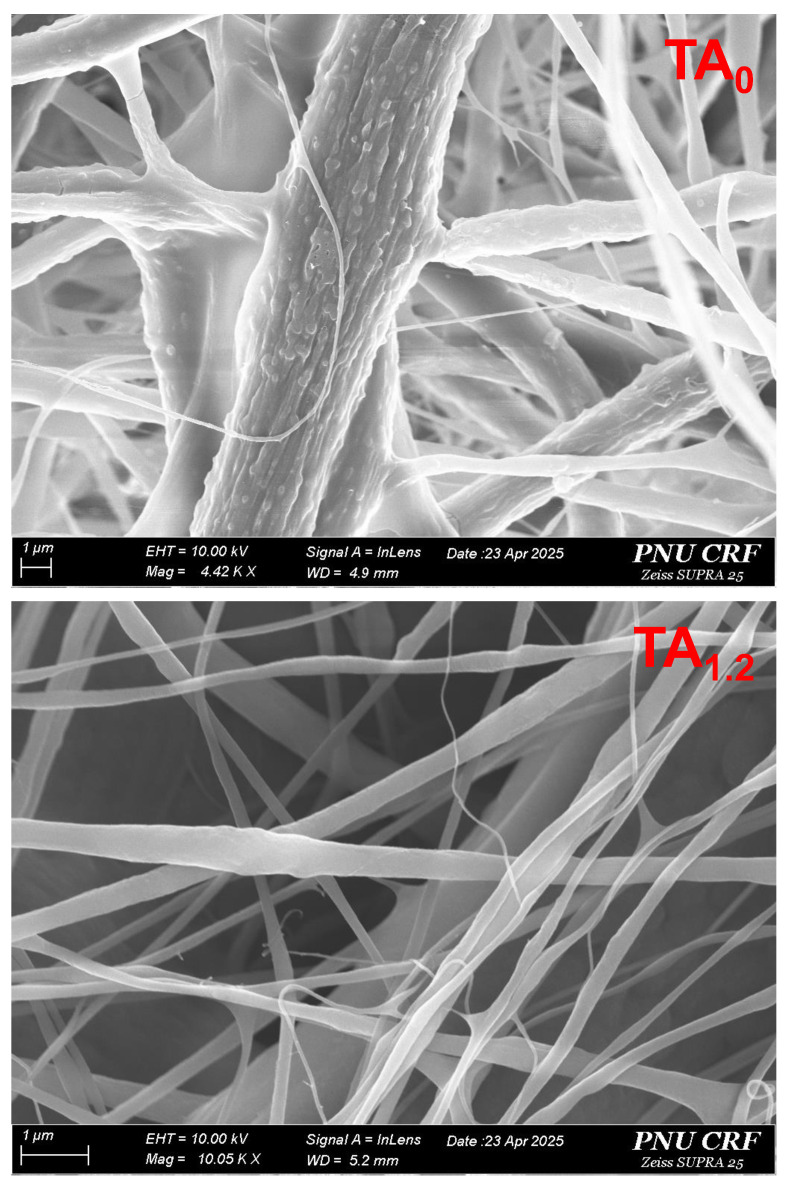
SEM images of NFs (TA_0_ and TA_1.2_).

Figure 1 presents SEM images that provide clear visual confirmation of the NF surface morphology [25]. The images reveal that the electrospun NFs are generally continuous with relatively consistent morphology, although some variability in diameter was observed (Figures S1 and S2). This bead-free morphology indicates successful optimization of the electrospinning parameters, such as solution concentration, voltage, and flow rate. The NFs exhibit desirable characteristics, including being non-aggregated, flexible, soft, homogeneous, and easy to handle, all of which are critical for practical application in flexible NFs. Table S1 summarizes the key physicochemical properties of the electrospinning solutions, including viscosity, conductivity, fiber shape, and average fiber diameter (AFD). These parameters significantly influence the morphology and quality of the resulting NFs. When TA was incorporated into the PVDF matrix, the conductivity of the spinning solution showed a slight increase, while its viscosity was somewhat reduced. Despite these changes, the overall differences in conductivity, viscosity, and AFD among the PVDF and TA NFs were not statistically significant. Interestingly, the TA_0_ NFs exhibited a slightly larger AFD compared to those containing TA (PVDF/TA). This could be attributed to the altered solution properties induced by TA addition, specifically, the combined effect of reduced viscosity and marginally increased conductivity. Typically, higher solution viscosity leads to thicker fibers, while higher conductivity promotes fiber stretching, yielding finer diameters. In the case of the PVDF/TA solution, the slight reduction in viscosity may have allowed the polymer jet to elongate more easily under the electric field, leading to a modest reduction in AFD. These results suggest that the incorporation of TA does not negatively impact the structural integrity or quality of the NFs and may even contribute to slight improvements in fiber uniformity and manageability.

##### DSC Analysis

**Figure 2:**
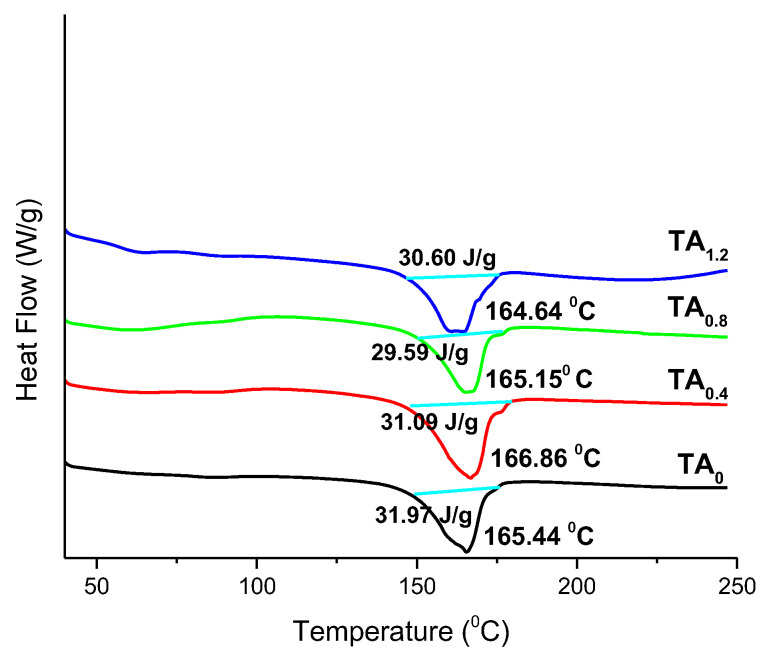
DSC of prepared NFs.

DSC is a widely employed thermal analysis technique used to investigate the thermal behavior and phase transitions of polymer-based NFs, particularly focusing on their melting point temperature (T_m_) [26]. This technique offers valuable insights into the thermal stability, crystallinity, and purity of NF materials, making it an essential tool for characterizing their thermal properties [27]. Figure 2 presents the DSC thermograms of the NFs incorporated with TA. For the pristine PVDF (TA_0_) NFs, a distinct endothermic peak corresponding to the Tm was observed at 165.44 °C [27], which serves as a reference for comparison. Upon the incorporation of TA, a slight decrease in the melting temperature was noted in the DSC profiles of the modified NFs, indicating a minimal interaction between TA and the PVDF matrix that marginally affects the crystalline structure. Furthermore, the enthalpy of fusion (heat of melting, ΔHf) remains nearly unchanged across all NF samples, with values consistently around 30 J/g. This negligible variation suggests that the overall thermal stability and crystallinity of the NFs were retained despite the presence of TA, indicating good compatibility and structural integrity of the composite system at elevated temperatures [28,29].

##### Stability Aspects with DSC Analysis

The thermal stability of the NFs is a critical parameter for assessing their performance under elevated temperature conditions, especially for applications in food packaging, biomedical scaffolds, or filtration systems. DSC analysis revealed that the incorporation of TA into the PVDF NFs induces only a slight reduction in the T_m_, suggesting minimal disruption to the crystalline regions of the polymer. The T_m_ values of the TA-modified NFs remain close to that of PVDF (165.44 °C), indicating that the PVDF backbone retains its thermal resistance despite the presence of TA. More importantly, the ΔH_f_, a measure of crystallinity and thermal energy required for phase transition, remains nearly constant (~30 J/g) across all NF samples. This constancy implies that the degree of crystallinity and the internal ordering of the polymer chains are preserved, and no significant thermal degradation or phase instability occurs due to TA incorporation [30]. The stable thermal behavior demonstrates that the polymer network can maintain its integrity under thermal stress, making the material suitable for applications requiring moderate to high thermal endurance. The results also confirm a high degree of compatibility between TA and the PVDF matrix, which supports long-term stability and reliability of the NFs. 

### 3.2. The Hydrophilic to Hydrophobic Nature of NFs for Release Behavior

The release behavior of electrospun NFs was significantly influenced by the physicochemical properties of the polymer employed. Factors such as hydrophilicity or hydrophobicity directly impact sample encapsulation efficiency and release kinetics. Typically, hydrophilic polymers promote faster drug release due to enhanced water uptake [31], while hydrophobic polymers tend to impede water diffusion, enabling a more controlled and sustained release profile [32]. In the present study, PVDF was selected as the base polymer due to its highly hydrophobic and semicrystalline nature. This inherent hydrophobicity plays a pivotal role in modulating drug release by forming a barrier that limits water penetration into the fiber matrix, thereby slowing the diffusion of the encapsulated drug. Specifically, in the case of the hydrophilic sample TA, the hydrophobic environment created by PVDF hinders immediate diffusion and reduces its mobility within the matrix. Additionally, hydrophobic interactions between PVDF and TA further contribute to sustained drug release by minimizing burst effects and enhancing release uniformity. The use of PVDF facilitates a prolonged release profile, which was particularly advantageous for delivering hydrophilic drugs in a controlled manner.

### 3.3. Antifungal and Antibiofilm Efficacy of NFs

**Figure 3:**
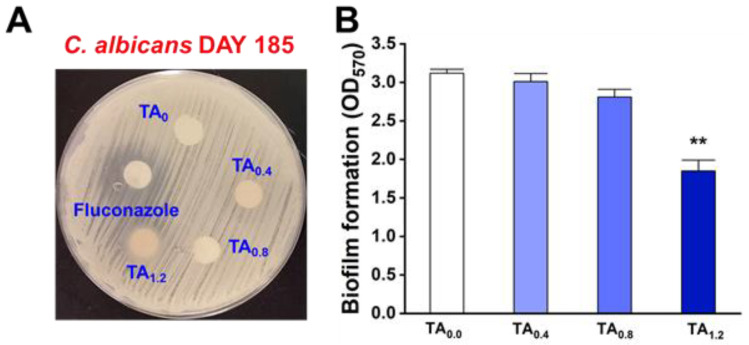
Evaluation of antifungal activity of TA_0_, TA_0.4_, TA_0.8_, TA_1.2_, and fluconazole (Positive control) against *C. albicans* DAY 185 (**A**), and antibiofilm potency (**B**) of TA_0_, TA_0.4_, TA_0.8_, TA_1.2_, and fluconazole (Positive control) against *C. albicans* DAY 185.

The antifungal susceptibility of *C. albicans* to TA-loaded NFs was confirmed through an agar disc diffusion assay. Among the tested samples, the TA_1.2_ NFs exhibited the most pronounced antifungal activity, with a clear inhibition zone visible around the disc. Representative images of the inhibition zones are presented in Figure 3A. Specifically, NFs with TA_0.8_ and TA_1.2_ produced inhibition zones measuring 0.2 ± 0.1 mm and 7.2 ± 1.1 mm, respectively (excluding the 9 mm disc diameter). In contrast, no antifungal activity was observed for the NFs with TA_0_ and TA_0.4_ (Table S2). The antibiofilm potential of the NFs was further assessed using a *C. albicans* biofilm inhibition assay. As shown in Figure 3B, NFs with TA_1.2_ significantly suppressed biofilm formation after 24 h of incubation. At this concentration, biofilm biomass was reduced by over 40 ± 4.5%, without affecting cell proliferation. NFs with TA_0.8_ also inhibited biofilm formation, achieving a reduction of more than 10 ± 3.1%. In contrast, NFs containing lower TA concentrations, such as TA_0.4_, demonstrated minimal biofilm inhibition (9.1 ± 0.2%), while TA_0_ showed no detectable effect. These findings suggest that the antifungal and antibiofilm efficacy of the NFs was strongly dose-dependent, with higher concentrations of TA resulting in significantly enhanced inhibition of *C. albicans*. Furthermore, the final microbial load was quantitatively assessed using the colony-forming unit (CFU) method, which provides a reliable measure of viable microbial cells per gram of sample. The results indicated that there was no statistically significant difference in microbial counts between the samples covered with LDPE and PVDF NFs when compared to the uncovered control group. However, a notable exception was observed in the group covered with the TA_1.2_ NFs. This group demonstrated a substantial reduction in microbial load, with final counts averaging approximately 3.57 ± 0.58 log CFU/g, in contrast to the uncovered group, which showed significantly higher counts of about 6.3 ± 0.47 log CFU/g (Figure S3). This marked decrease in viable microbial cells in the TA_1.2_ group aligns closely with the findings from the antimicrobial activity assays, reinforcing the NFs’ enhanced efficacy in suppressing microbial growth.

### 3.4. Antioxidant (AO) Analysis

**Figure 4:**
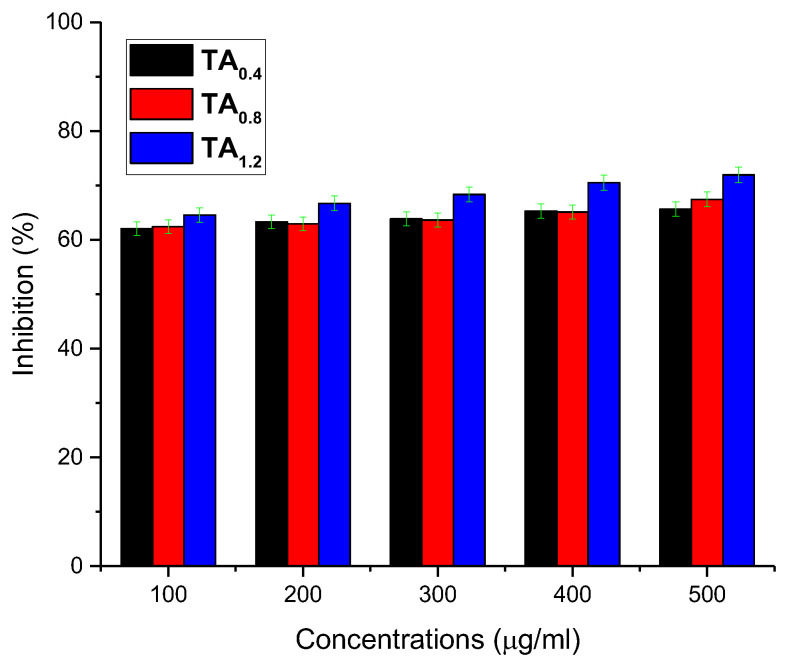
Antioxidant activity of NFs by DPPH assay.

The AO activity of the fabricated NFs was evaluated using the DPPH free-radical scavenging assay, a widely used method for assessing the ability of compounds to neutralize free radicals [29]. In this study, ascorbic acid was employed as a standard reference antioxidant to benchmark the performance of the prepared NFs. The samples, including three different NFs and the standard, were tested at varying concentrations, 100, 200, 300, 400, and 500 μg/mL, to observe the dose-dependent AO activity. The results, presented in Figure 4, illustrate the comparative antioxidant responses of all samples across the tested concentration range. All NFs demonstrated moderate radical scavenging activity, with increasing effectiveness observed at higher concentrations. Notably, the NFs labeled TA_1.2_, which contain the highest concentration of TA, exhibited significantly enhanced antioxidant activity relative to the other NF samples (as detailed in Table S3). This superior performance was attributed to the increased availability of phenolic hydroxyl groups from TA, which are known to donate hydrogen atoms to neutralize DPPH radicals effectively. The findings indicate that the incorporation of higher concentrations of TA into the NF matrix markedly improves the AO potential of the NFs. This enhancement suggests that TA_1.2_ NFs possess excellent radical scavenging properties, making them promising candidates for applications in active food packaging, where antioxidant functionality is crucial for extending shelf life and maintaining product quality.

### 3.5. In Vitro Biocompatibility

**Figure 5:**
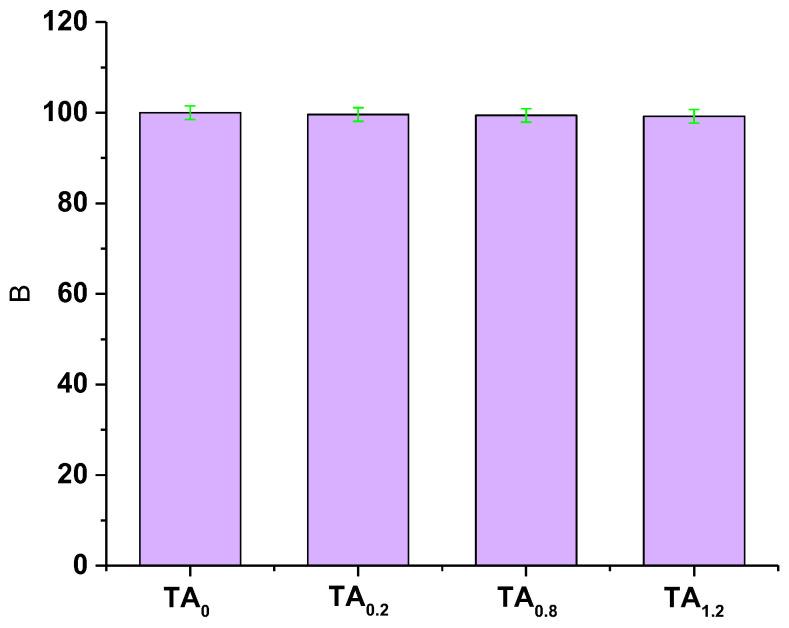
The cytotoxicity of TA_0_, TA_0.4_, TA_0.8_, and TA_1.2_ NFs on fibroblast cells.

**Figure 6:**
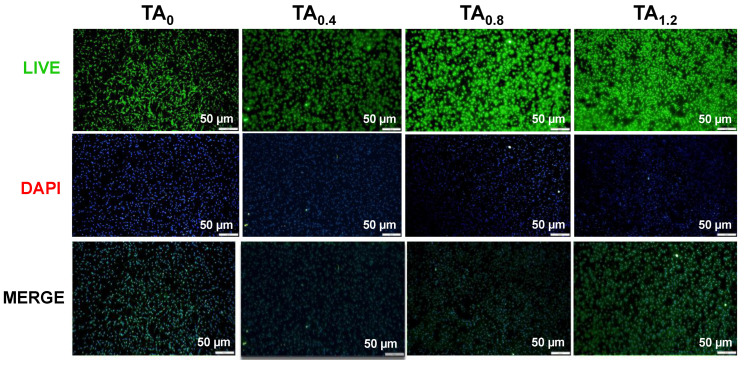
Live/dead fluorescence staining assay.

The cytotoxicity evaluation of TA_0_, TA_0.4_, TA_0.8_, and TA_1.2_ NFs revealed that none of these groups exhibited any toxic effects on fibroblast cells, as illustrated in Figure 5. The fibroblast cells maintained healthy morphology and proliferation across all tested samples, indicating that the TA_0_, TA_0.4_, TA_0.8_, and TA_1.2_ NF matrices were likely biocompatible and capable of supporting normal fibroblast cell growth without inducing cytotoxic responses. To further assess the biocompatibility and investigate the potential induction of apoptosis or necrosis, an acridine orange/ethidium bromide (AO/EtBr) staining assay was performed. Acridine orange selectively stains viable cells, producing a bright green fluorescence, while early apoptotic cells also fluoresce green but with higher intensity. In contrast, late apoptotic cells emit orange fluorescence, and necrotic or nonviable cells take up ethidium bromide, displaying red fluorescence. The staining results are presented in Figure 6. Observations from the AO/EtBr staining confirmed that fibroblast cells cultured on TA_0_, TA_0.4_, TA_0.8_, and TA_1.2_ NFs predominantly exhibited bright green fluorescence, characteristic of healthy, viable cells. No signs of early or late apoptosis, nuclear condensation, or necrosis were detected across any of the samples. Collectively, the results from the cytotoxicity assays strongly suggest that TA_0_, TA_0.4_, TA_0.8_, and TA_1.2_ NF scaffolds are cytocompatible. They do not provoke any toxic responses in fibroblast cells, thus affirming their potential suitability for biomedical applications where maintaining cell viability and function is critical.

### 3.6. Determination of Apoptosis by Cell Cycle Analysis

**Figure 7:**
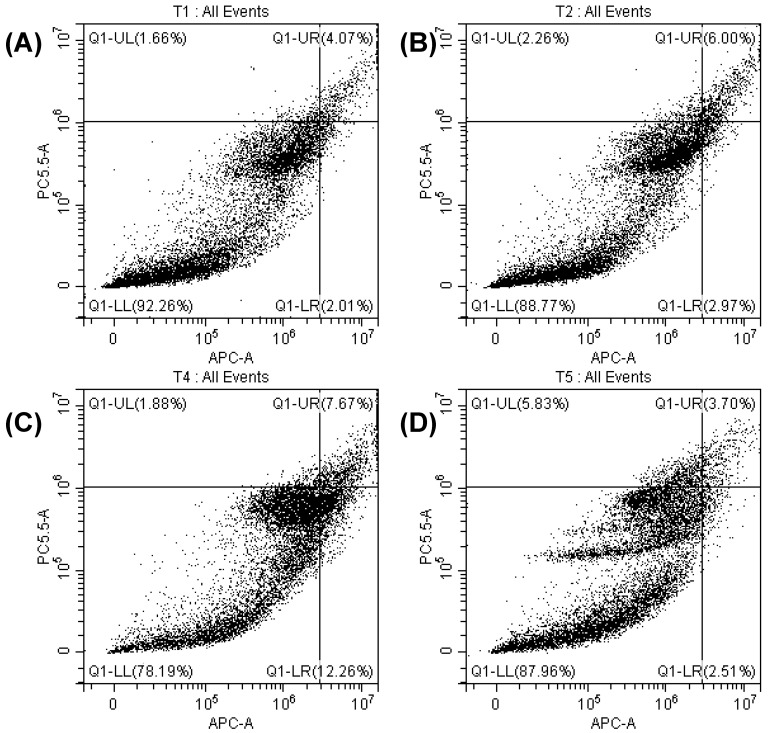
FACs of TA_0_ (**A**), TA_0.4_ (**B**), TA_0.8_ (**C**), and TA_1.2_ (**D**).

Apoptosis is a tightly regulated and systematic cellular process that involves distinct morphological and biochemical changes, such as chromatin condensation, nuclear fragmentation, cell shrinkage, and membrane blebbing. These hallmark features distinguish apoptotic cell death from necrosis, which is often associated with uncontrolled cell lysis and inflammation [33,34]. To investigate whether the fibroblast cells treated with TA_0_, TA_0.4_, TA_0.8_, and TA_1.2_ NFs underwent apoptosis or necrosis, flow cytometry analysis using Annexin V/7-Aminoactinomycin D (7AAD) staining was performed. Annexin V binds to phosphatidylserine residues translocated to the outer leaflet of the plasma membrane during early apoptosis, whereas 7AAD permeates and stains the DNA of late apoptotic and necrotic cells. This dual-staining approach allows clear discrimination between viable, early apoptotic, late apoptotic, and necrotic cell populations. Representative flow cytometry plots of fibroblast cells treated with TA_0_, TA_0.4_, TA_0.8_, and TA_1.2_ NFs are shown in Figure 7. The results demonstrated that fibroblast cells treated with TA_0_, TA_0.4_, TA_0.8_, and TA_1.2_ NFs exhibited minimal to no staining for Annexin V and 7AAD, indicating the absence of significant apoptotic or necrotic populations. The treated groups and the untreated control group displayed a predominant population of viable cells with intact membranes, suggesting that none of the treatments induced apoptotic or necrotic cell death under the experimental conditions. These findings clearly indicate that exposure to TA_0_, TA_0.4_, TA_0.8_, and TA_1.2_ NF matrices does not trigger programmed cell death in fibroblast cells. The consistency in viability across all samples further reinforces the cytocompatibility and non-cytotoxic nature of these materials.

### 3.7. Food-Packaging Analysis

#### 3.7.1. Water-Contact Angle

**Figure 8:**
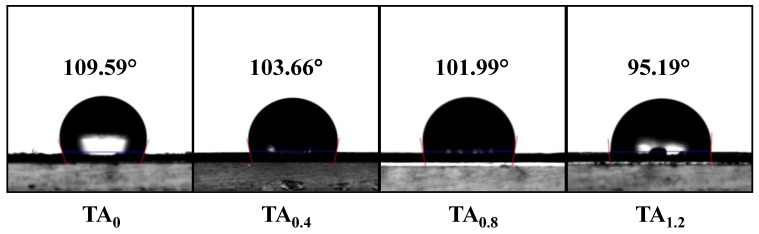
Water-contact angle images of TA_0_, TA_0.4_, TA_0.8_, and TA_1.2_ NFs.

The TA_0_ NFs exhibited a high contact angle, confirming their intrinsic hydrophobicity. This suggests that the surface of PVDF fibers resists wetting, characteristic of its non-polar nature. However, a slight decrease in the contact angle was observed as the concentration of TA increased in the composite material. This reduction in contact angle can be attributed to the interactions between the hydroxyl groups of TA and the PVDF surface, which may enhance the surface’s ability to attract moisture to a minor extent (Figure 8). Despite this slight decrease, the electrospun PVDF and its composites maintain strong hydrophobicity overall. The results indicate that these materials retain their moisture-repellent properties, which is a key factor for their potential use as effective moisture-barrier packaging materials in food applications. The high hydrophobicity ensures that the materials can effectively prevent water-vapor penetration, thus preserving the quality and shelf life of food products.

#### 3.7.2. Thermal Shrinkage

**Figure 9:**
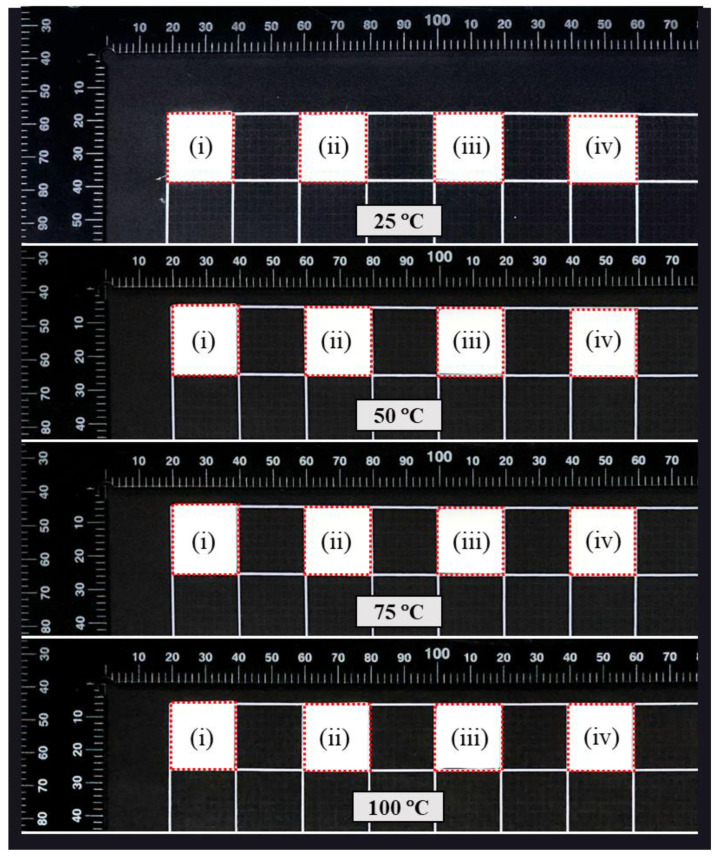
Thermal shrinkage behavior of composite films: (**i**) TA_0_, (**ii**) TA_0.4_, (**iii**) TA_0.8_, and (**iv**) TA_1.2_ at varied temperatures (25 °C, 50 °C, 75 °C, and 100 °C).

No shrinkage was observed in any of the films across the temperature ranges tested, indicating excellent dimensional stability. This lack of shrinkage, even under elevated temperatures, suggests that the films retain their shape and structure, which was a critical factor for their performance in practical applications. The absence of dimensional change under thermal stress reflects the robustness of the materials, particularly their resistance to deformation when exposed to heat (Figure 9). This thermal stability was particularly significant for packaging applications where the films may come into contact with hot food items or be subjected to varying temperatures during storage and transportation. The ability to maintain integrity under heat ensures that these films can effectively protect food products without losing their form or functionality, making them highly suitable for use in packaging solutions for hot or temperature-sensitive foods.

#### 3.7.3. Packaging Study

**Figure 10:**
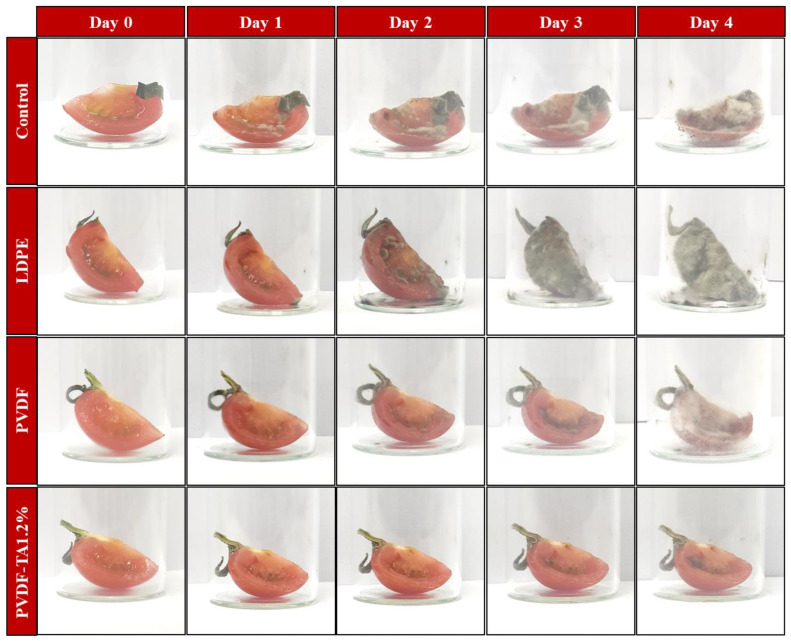
Visual appearance of fresh-cut cherry tomatoes stored under different packaging conditions (control, LDPE, TA_0_, and TA_1.2_) over 4 days.

The TA_1.2_ NFs exhibited a slower rate of microbial spoilage compared to the control, low-density polyethylene (LDPE) and TA_0_ NFs. This enhanced performance suggests that the incorporation of TA at a 1.2% concentration significantly contributes to the film’s antimicrobial properties. The slower microbial growth observed in TA_1.2_ indicates that TA acts as an effective preservative, likely due to its known antimicrobial and antifungal characteristics (Figure 10). TA, with its polyphenolic structure, may interact with microbial cell membranes, disrupting their integrity and inhibiting growth. In contrast, the control and the pure PVDF films, which lack such antimicrobial agents, demonstrated more rapid microbial spoilage. The LDPE film, while commonly used in food packaging, showed no significant advantage in microbial resistance compared to the PVDF-based films, highlighting the added benefit of incorporating TA into PVDF for improved shelf life and food preservation. This suggests that TA_1.2_ could be a promising candidate for food-packaging applications, particularly in extending the freshness and quality of food products by reducing microbial contamination [35].

#### 3.7.4. Weight Loss

**Figure 11:**
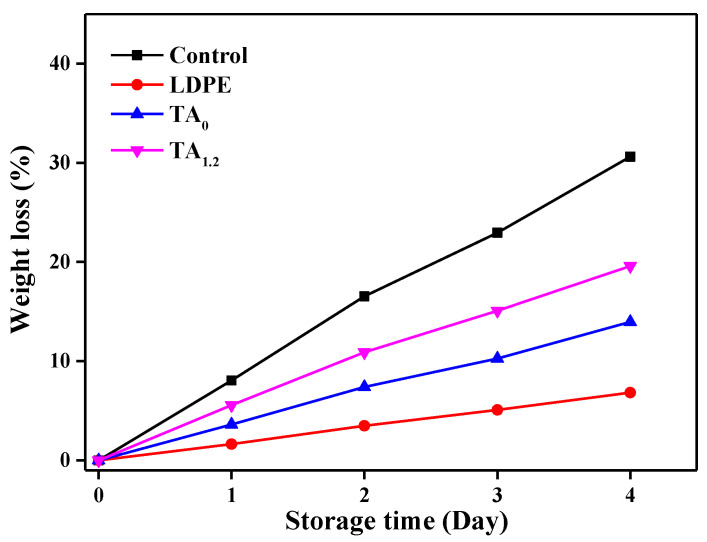
Weight loss (%) of fresh-cut cherry tomatoes over 4 days of storage under different packaging conditions: control (no packaging), LDPE, TA_0_, and TA_1.2_.

The control group exhibited the highest weight loss (30.62%) at the end of the 4-day packaging study, which can be attributed to direct exposure to the surrounding storage environment. This significant weight loss was likely a result of moisture absorption and the overall instability of the material under the given conditions. Among the different film packaging materials tested, LDPE demonstrated the least weight loss (6.82%), indicating its excellent moisture-barrier properties. LDPE’s dense structure effectively prevents the diffusion of water vapor, which helps in maintaining its integrity and minimizing weight loss during exposure to humid or fluctuating environmental conditions. In contrast, both electrospun TA_0_ and TA_1.2_ composites experienced higher weight loss (13.96% and 19.59%, respectively) during the same 4-day period (Figure 11). This can be attributed to their inherently porous structure, which, while beneficial for certain applications, allows for greater water-vapor permeability [36]. The porosity of electrospun PVDF provides more pathways for moisture to migrate through the material, thus increasing the likelihood of weight loss over time. Additionally, the incorporation of TA in the TA_1.2_ composite may further influence its water absorption characteristics, potentially enhancing its permeability to moisture. Although we did not perform WVP tests, the observed weight loss in these films suggests that their increased water-vapor transmission rate could be a contributing factor. This highlights a trade-off between the material’s moisture permeability and its overall weight stability, which should be carefully considered depending on the specific application of the packaging material. Despite the higher weight loss in TA_0_ and TA_1.2_, their other functional properties, such as antimicrobial performance, may still make them suitable for certain types of food packaging where moisture control is less critical. 

#### 3.7.5. Comparison of Food Preservation with the Literature

In recent years, significant progress has been made in the development of NF-based materials enriched with active antifungal and antibacterial agents for food preservation. These advanced materials have been successfully applied to perishable fruits such as kiwis, cherry tomatoes, and strawberries, demonstrating remarkable potential in extending shelf life and preventing microbial spoilage [1,5,37]. The innovative structural design of nanofibers (NFs) has played a crucial role in advancing the field of active food packaging, providing a robust platform for the sustained and controlled release of bioactive compounds [7]. Building on these advancements, our study presents a novel application of NF-based packaging, specifically designed for the preservation of tomatoes. The results revealed that the NF system effectively inhibited fungal growth throughout the storage period without inducing any detectable toxicity. This finding marks a significant breakthrough in the area of food-preservation technologies, offering a safe and efficient method to enhance the post-harvest stability of tomatoes.

## 4. Conclusions

In conclusion, this study successfully developed bioactive NF-based materials composed of TA within a PVDF matrix, demonstrating their potential as an effective active packaging solution for the preservation of cherry tomatoes. The incorporation of TA at concentrations (TA_1.2_) into the PVDF matrix resulted in NFs with promising physicochemical properties, including moisture content, opacity, and thermal stability, which were essential for maintaining the quality of fresh produce. The most notable antifungal and antibiofilm characteristics were demonstrated by the TA-loaded PVDF NFs (TA_1.2_), which successfully stopped fungal damage to the cherry tomatoes and reduced total microbial counts. Shelf-life tests revealed that tomatoes coated with these NFs exhibited no visible surface changes for up to 4 days, highlighting the potential of these bioactive films in extending the shelf life of perishable food items. Furthermore, the non-toxic nature of the NFs to normal cells, as confirmed by cytotoxicity tests, supports their safe use in food-packaging applications. Hence, the developed TA NFs provide a sustainable and effective alternative to conventional synthetic packaging materials, offering a promising solution for food preservation via enhancing shelf life while contributing to food safety and sustainability.

## 5. Study Limitations

While the developed TA NFs demonstrated promising results in enhancing the shelf life of cherry tomatoes, the study has certain limitations that warrant further investigation. First, the shelf-life extension observed was limited to up to 4 days, which, although beneficial, may not be sufficient for broader commercial applications. Future studies should explore ways to further enhance the longevity of the preservative effect, potentially by combining TA with other natural bioactive compounds or optimizing the electrospinning process to improve barrier properties. Second, the study focused exclusively on cherry tomatoes as a model perishable item. To validate the versatility of the developed NFs, similar preservation studies should be conducted on a wider variety of fruits and vegetables with different respiration rates and susceptibility to spoilage. Another limitation was the absence of a real-time microbial analysis to quantify bacterial or fungal growth over the storage period. Incorporating quantitative microbiological assessments will provide deeper insight into the antimicrobial mechanisms and efficacy of the TA-loaded NFs.

## 6. Future Scope of the Current Study

In future directions, integrating smart packaging features such as spoilage indicators, enhancing mechanical strength for industrial handling, and developing multi-layered structures for tailored performance could broaden the applicability of TA/PVDF NFs in active food packaging. Additionally, incorporating hydrophobic surface coatings, barrier-layer laminations, or moisture-resistant additives could help reduce water-vapor transmission without compromising antimicrobial functionality. These enhancements will contribute to the development of next-generation sustainable packaging solutions that align with global food safety and environmental goals.

## Data Availability

The raw data supporting the conclusions of this article will be made available by the authors on request.

## Supplementary Materials

The following supporting information can be downloaded at: https://www.mdpi.com/article/10.3390/ma18133112/s1, Scheme S1. Electrospinning process of PVDF and TA NFs; Figure S1. Photos of prepared NFs; Figure S2. SEM images of NFs (TA_0_ and TA_1.2_); Figure S3. The effect of LDPE, TA_0_, and TA_1.2_ on the total microbial count of fresh-cut cherry tomatoes after 4 days; Table S1. Properties of the electrospinning solution and outputs; Table S2. Antifungal efficacy of TA_0.0_, TA_0.4_, TA_0.8_, and TA_1.2_ against *C. albicans* DAY 185 by a zone of inhibition (after subtracting NF disc diameter 9 mm value); Table S3. Antioxidant performances of NFs with respect to different concentrations; Table S4. Color parameters (L*, a*, b*) and total color difference (ΔE) of NFs; Table S5. Thickness and mechanical properties of NFs. References [38,39,40,41,42,43] are cited in the Supplementary Materials. The details of biocompatibility, characterization techniques, experimental procedures of anti-fungal and anti-biofilm, packaging applications, and their important parameters, such as water contact angle, mechanical properties, and thermal shrinkage.
