# Supplementary material for: Electrospun Nanofibers of Polyvinylidene Fluoride Enriched with Active Antimicrobial Tannic Acid for the Improvement of the Shelf Life of Cherry Tomatoes
Source: Materials (Basel). 2025 Jul 1;18(13):3112. doi: 10.3390/ma18133112 (PMC12250959; doi:10.3390/ma18133112)
Supplement: Supplementary file 1 [file materials-18-03112-s001.zip › materials-3662478 supplementary.pdf]

Electrospun nanofibers of polyvinylidene fluoride enriched with active anti-microbial tannic acid for the improvement of the shelf life of cherry tomatoes

Rajaram Rajamohan[a*], Ajmal P Muhammed[a], Chaitany Jayprakash Raorane[a], Subramaniyan Ramasundaram[a], Iruthayapandi Selestin Raja[b], Sivakumar Allur Subramanian[c, d], Seong-Cheol Kim[a], Tae Hwan Oh[a], Seho Sun[a*]

[a] School of Chemical Engineering, Yeungnam University, Gyeongsan 38541, Republic of Korea.

[b]Institute of Nano-Bio Convergence, Pusan National University, Busan, 46241 Republic of Korea.

[c] Department of Orthopaedic Surgery, Dongtan Sacred Heart Hospital, Hallym University, College of Medicine, Hwaseong, Republic of Korea.

[d] Department of Orthopedics & Rehabilitation, Carver College of Medicine, University of Iowa, Iowa City, IA 52242.

***Correspondence: rajmohanau@yu.ac.kr (R.R); seho.sun@yu.ac.kr (S.S)**

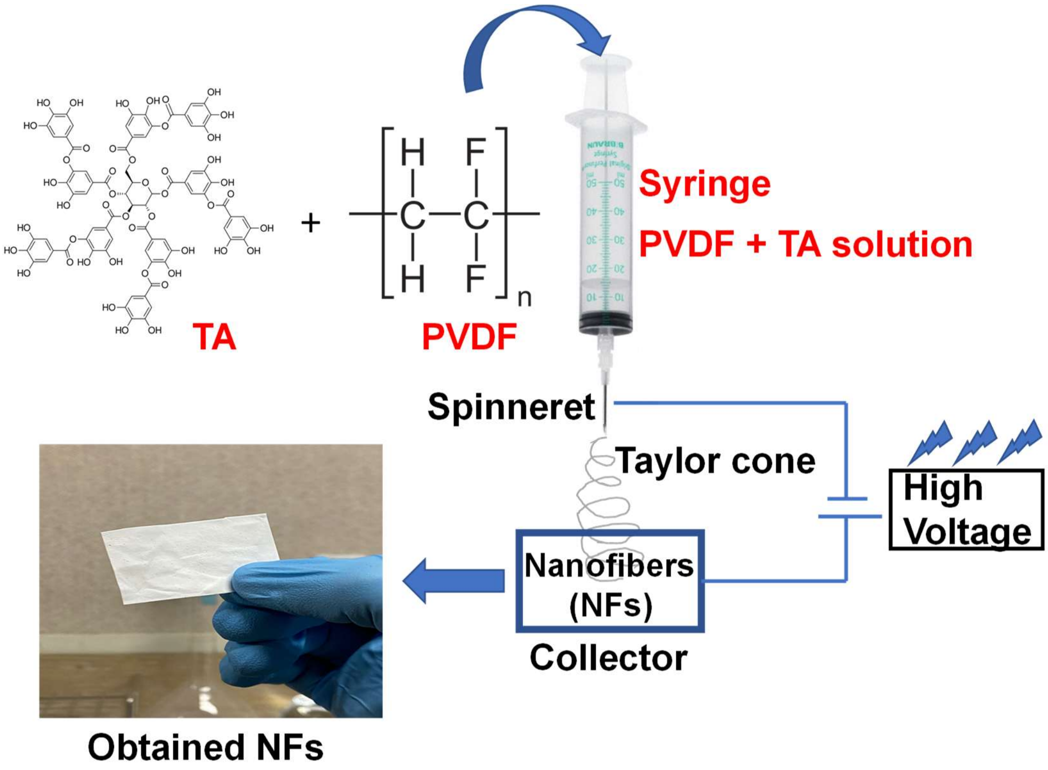

Scheme S1. Electrospinning process of PVDF and TA NFs **[S1]**.

Cell culture

Mouse 3T3-L1 fibroblast cells were purchased from the American Type Culture Collection (Manassas, VA). Briefly, the cells were grown in a 75 cm^2 culture flask with DMEM containing 10 % FBS and 1 % penicillin/streptomycin at 37 °C in 5 % CO_2. The fibroblast cells were expanded without differentiation in 25-cm^2 culture flasks at a density of 1.0×10^4 cells/cm^2. Every three days, the medium was changed. After reaching 80–90% confluence, the cells were harvested from the sub-confluent monolayer after their detachment by exposure to trypsin containing 5.3 mM EDTA at 37°C for 2 min and gently tapped with an open palm to release the fibroblasts and aspirate the trypsin-EDTA. This was added to a 15-mL centrifuge tube already containing 5 mL of DMEM + FBS solution and centrifuged. After centrifugation, the pellets were collected and seeded (1:10 ratio) into new culture flasks.

Cell metabolic activity (MTT) assay on the PVDF/TA

Experimental groups

Group 1: TA_0 NFs

Group 2: Fibroblast cells treated with $TA_{0.4}$ NFs

Group 3: Fibroblast cells treated with $TA_{0.8}$ NFs

Group 4: Fibroblast cells treated with $TA_{1.2}$ NFs

Mitochondrial activity tests by trypan blue staining

Cytotoxicity and cell viability of nanofibers were measured by 2% trypan blue staining **[S2]**. The number of viable cells was estimated in each group by counting in a Neubauer chamber.

Cell attachment (live/dead) assay on the TA_0, $TA_{0.4}$, $TA_{0.8}$, and $TA_{1.2}$ NFs

Fibroblast cells (2×10^5 cells/well) were grown on TA_0, $TA_{0.4}$, $TA_{0.8}$, and $TA_{1.2}$ NFs for 24h. AO/EtBr staining was performed to detect condensed chromatin of dead apoptotic cells **[S3]**. Apoptotic cells will uptake EtBr and emit red/orange fluorescence under 550 nm. Acridine orange is a DNA-selective and membrane-permeable fluorescent cationic dye that freely enters normal cell nuclei and emits green fluorescence at 525 nm. During apoptosis, DNA becomes condensed and fragmented. Stained cells were viewed under a fluorescence microscope LSM510META (Carl Zeiss, Jena, Germany).

Determination of apoptosis by FACS analysis

According to the manufacturer's instructions, the FITC Annexin V Apoptosis Detection Kit with 7-AAD was utilized to determine the apoptosis and necrosis in TA_0, $TA_{0.4}$, $TA_{0.8}$, and $TA_{1.2}$ NFs for 24 h-treated cells. After the incubation period, the collected cells were washed with PBS, then suspended in Annexin V binding buffer, and incubated for 15 min in the dark with FITC-labeled Annexin V and 7AAD. Flow cytometry evaluated the samples right away (BD, Franklin Lakes, NJ, USA).

Instruments Used

Field Emission Scanning Electron Microscopy (FE-SEM) analysis was performed using a Hitachi S-4800 microscope operated at an accelerating voltage of 10.0 kV to examine the surface morphology of the NFs. The thermal behavior of the NFs was evaluated using a Differential Scanning Calorimeter (DSC) from TA Instruments. Thermal analysis curves were generated and interpreted using the Universal Analysis software version 4.5A. For each DSC measurement, approximately 5.25 mg of NFs was used, and the temperature was ramped from 40.0 °C to 250.0 °C at a constant heating rate of 10 °C/min under a nitrogen atmosphere. DSC were conducted at the Core Research Support Center (CRSC) for Natural Products and Medical Materials, Yeungnam University, South Korea.

In Vitro Evaluation of TA NFs Against Candida albicans

The *Candida albicans* strain DAY185 was obtained from the Korean Culture Center of Microorganisms (KCCM, South Korea; http://www.kccm.or.kr). The strain was maintained and sub-cultured on Potato Dextrose Agar (PDA) or in Potato Dextrose Broth (PDB).

Biofilm Inhibition Assay

Biofilm formation assays were performed following established protocols **[S4]**. Briefly, an overnight culture of *C. albicans* grown in PDB was used to assess the antibiofilm activity of NFs containing varying concentrations of TA: TA_0, $TA_{0.4}$, $TA_{0.8}$, and $TA_{1.2}$. Circular NFs discs (0.5 cm diameter) were prepared and placed in individual wells of a 96-well plate. The wells were inoculated with the fungal suspension and incubated at 37°C for 24 hours under static conditions to promote biofilm development on the nanofiber surfaces. Following incubation, biofilms were stained with 0.1% crystal violet for 20 minutes. Excess stain was removed, and the bound crystal violet was solubilized in 95% ethanol. Absorbance at 570 nm (OD_{570}) was measured to quantify the total biofilm mass on each sample.

Antifungal activity (Disc Diffusion Method) and total microbial count

The antifungal efficacy of the NFs was evaluated using the disc diffusion assay, as previously described **[S5]**. Single colonies of *C. albicans* were inoculated into PDB and adjusted to a 0.5 McFarland standard. Circular NFs discs (9 mm diameter) of TA_0, $TA_{0.4}$, $TA_{0.8}$, and $TA_{1.2}$ were prepared and sterilized under UV light for 15 minutes in a laminar flow cabinet.

Fluconazole (500 μg/mL) was used as positive control. PDA plates were uniformly swabbed with the standardized fungal suspension. Sterile nanofiber discs were placed onto the inoculated plates and incubated at 37°C for 24 hours. Zones of inhibition were measured in duplicates, and average diameters were calculated. All antifungal tests were conducted using at least three independent fungal cultures to ensure reproducibility, in accordance with Clinical Laboratory Standards Institute (CLSI) guidelines for yeasts (CLSI, 2017). Besides, the final microbial count was determined by the colony-forming unit (CFU) method with minor modifications **[S6]**. At the end of the packaging experiment (4 days), 0.5 g of each cherry tomato was removed and homogenized vigorously with 5 mL of sterile saline solution (0.85 % NaCl (w/v)) and then serially diluted to 10^6. Later, 100 μL of each dilution was separately spread onto Mueller-Hinton agar and incubated at 37°C for 24 h. The CFU of each sample was determined in triplicate and displayed as log CFU/g.

Experimental procedure for anti-oxidant activity by DPPH assay

The free radical scavenging activity was assessed using the DPPH assay, following a modified protocol based on Sivakumar et al. (2014) **[S7]**. DPPH is a stable nitrogen-centered free radical that exhibits a deep violet color in methanol, with a characteristic absorption peak at 517 nm. Upon interaction with antioxidant molecules capable of donating a hydrogen atom or an electron, DPPH is reduced to a non-radical form, resulting in a gradual color shift from violet to yellow. The degree of discoloration reflects the radical scavenging capacity of the tested compound. In this study, 1 mL of DPPH solution was combined with various concentrations (100, 200, 300, 400, and 500 μg/mL) of nanofiber formulations ($TA_{0.4}$, $TA_{0.8}$, and $TA_{1.2}$), and the volume was adjusted to 3 mL with distilled water. The mixtures were incubated at 37°C for 10 minutes, after which the absorbance was measured at 517 nm using a UV–Vis spectrophotometer. The decrease in absorbance indicated the extent of DPPH radical scavenging activity.

Color

The color of TA NFs was determined by a color meter (TES-135A, Taiwan). The instrument was calibrated using a standard white plate with values of L* = 93.42, a* = 1.395, and b* = 1.792 before measurement. Color measurements were made at least 3 randomly distributed points of each film sample against the white plate as background. The color values obtained were L* (brightness), a* (red–green axis), and b* (yellow–blue axis). The total color

difference (ΔE) between TA-added NFs and pure PVDF NFs (TA0) was calculated by the following equation (1):

$$\Delta E = \sqrt{(\Delta L)^2 + (\Delta a)^2 \pm (\Delta b)^2} \quad .... (1)$$

Mechanical properties

The thickness of films was determined by using a Mitutoyo digital micrometer (No. 293-240, Japan) with an accuracy of 0.001 mm. The thickness of samples was determined at 5 randomly chosen points, and then the average is reported. Tensile strength (TS) and elongation at break (EAB) were evaluated according to ASTM D882 using a universal testing machine (INSTRON 3345, USA). Film strips (10 mm × 50 mm) were tested at a crosshead speed of 10 mm/min with an initial grip separation of 30 mm. Each sample was tested in triplicate, and the results were averaged.

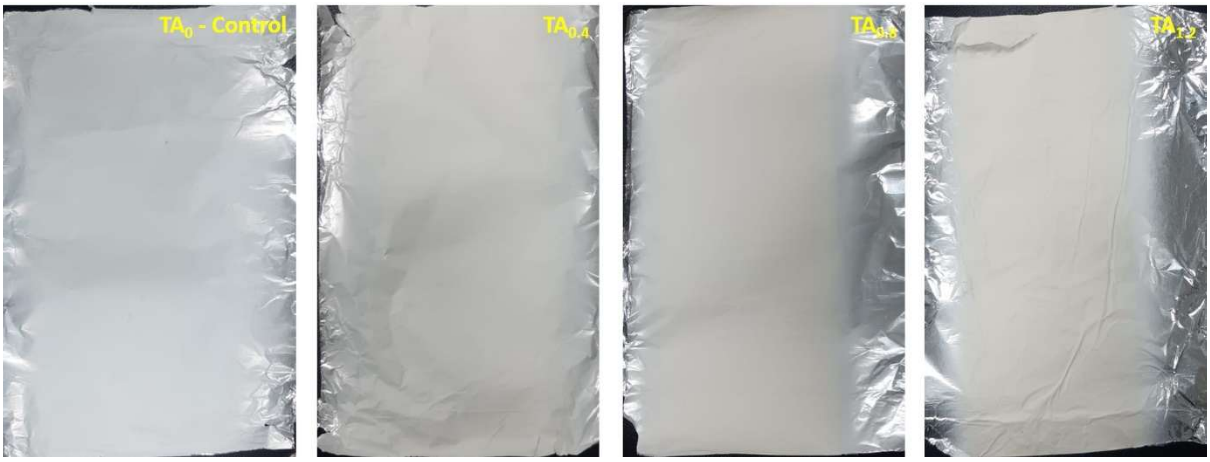

Figure S1. Photos of prepared NFs.

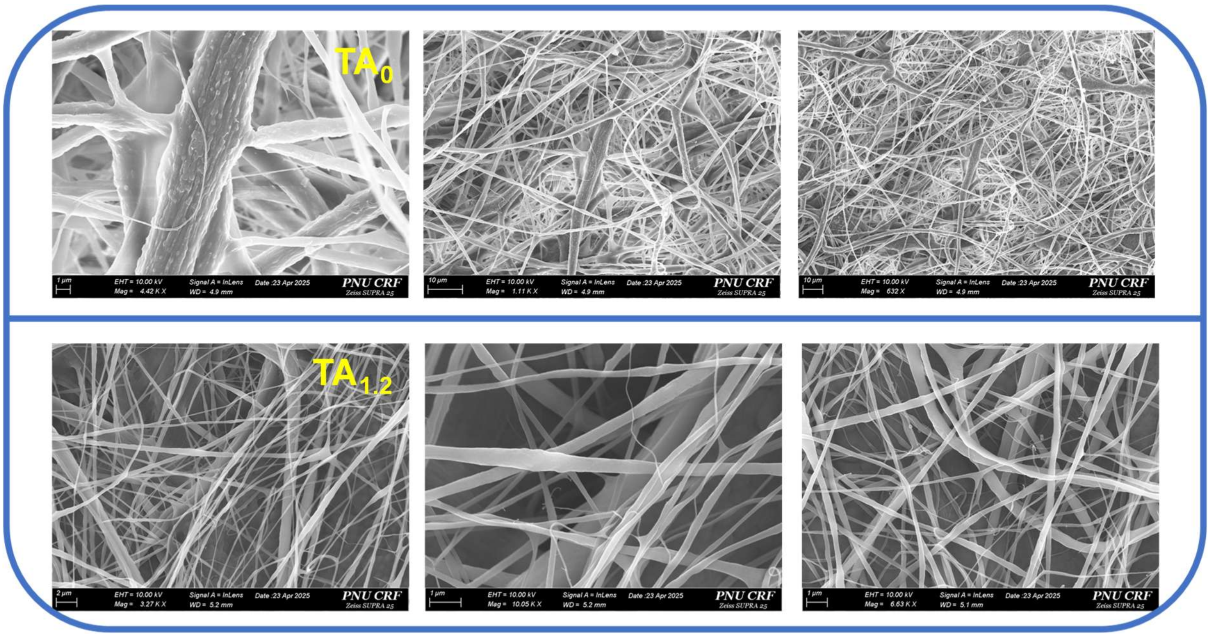

Figure S2. SEM images of NFs (TA_0, and $TA_{1.2}$).

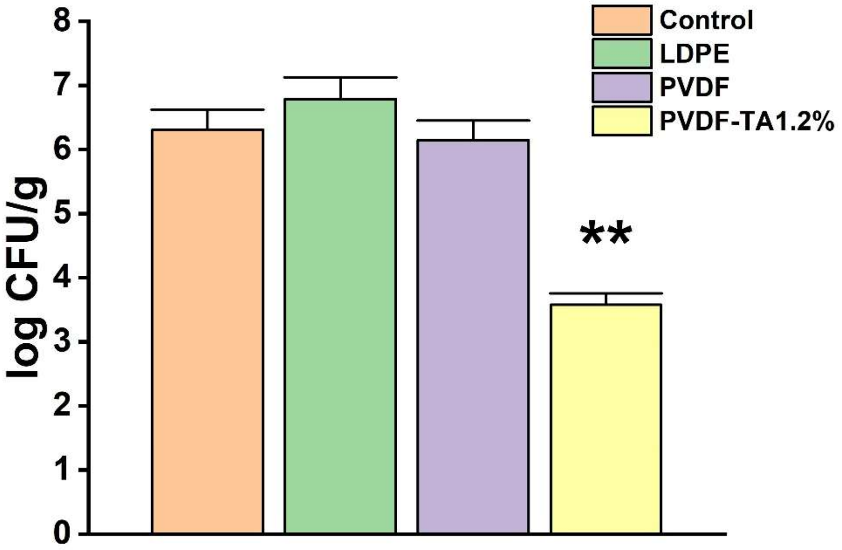

Figure S3. The effect of LDPE, TA_0, and $TA_{1.2}$ on the total microbial count of fresh-cut cherry tomatoes after 4 days.

Table S1. Properties of the electrospinning solution and outputs.

S. No	Solution	Viscosity	Morphology of NFs	Average fiber diameter (AFD), nm
**1.**	TA_0	0.18±0.01	Bead-free NFs	550±192
**2.**	$TA_{0.4}$	0.18±0.02	Bead-free NFs	615±122
**3.**	$TA_{0.8}$	0.18±0.15	Bead-free NFs	575±134
**4.**	$TA_{1.2}$	0.18±0.25	Bead-free NFs	618±148

* The measurements are repeated three times (n=3) and the average results are given with standard deviations.

Table S2. Antifungal efficacy of $TA_{0.0}$, $TA_{0.4}$, $TA_{0.8}$ and $TA_{1.2}$ against *C. albicans* DAY 185 by a zone of inhibition (After subtracting NFs discs diameter 9 mm value).

**Fungal strain**	**Zone of Inhibition (mm)**			
	All 4 NF have a diameter of 9 mm			
	$TA_{0.0}$	$TA_{0.4}$	$TA_{0.8}$	$TA_{1.2}$
C. albicans DAY 185	0.0 ± 0.0	0.0 ± 0.0	0.2 ± 0.1	7.1 ± 1.1

Table S3. Anti-oxidant performances of NFs with respect to different concentrations.

**S. No**	**Concentrations of NFs**	**$TA_{0.4}$**	**$TA_{0.8}$**	**$TA_{1.2}$**
1	100	62.05	62.41	64.56
2	200	63.30	62.94	66.72
3	300	63.84	63.66	68.34
4	400	65.28	65.11	70.50
5	500	65.64	67.44	71.94

Table S4. Color parameters (L*, a*, b*) and total color difference (ΔE) of NFs.

Sample	L*	a*	b*	ΔE
**TA_0**	94.85 ± 0.17^{a}	1.18 ± 0.41^{d}	0.97 ± 0.20^{c}	0
**$TA_{0.4}$**	94.09 ± 0.34^{b}	2.51 ± 0.38^{c}	5.70 ± 0.14^{b}	4.98 ± 0.40^{b}
**$TA_{0.8}$**	92.69 ± 0.57^{c}	3.23 ± 0.29^{b}	8.74 ± 1.45^{a}	8.33 ± 0.72^{a}
**$TA_{1.2}$**	91.75 ± 0.07^{d}	4.06 ± 0.29^{a}	9.73 ± 1.01^{a}	9.73 ± 1.13^{a}

Table S5. Thickness and mechanical properties of NFs.

Sample	Thickness (µm)	Tensile strength (MPa)	Elongation at break (%)
**TA_0**	90.20 ± 6.84^{c}	2.95 ± 0.50^{a}	5.28 ± 1.60^{a}
**$TA_{0.4}$**	102.80 ± 8.58^{c}	1.86 ± 0.13^{b}	5.08 ± 0.78^{a}
**$TA_{0.8}$**	134.80 ± 14.25^{b}	1.69 ± 0.19^{b}	4.28 ± 0.91^{a}
**$TA_{1.2}$**	203.80 ± 21.52^{a}	1.54 ± 0.11^{b}	1.34 ± 0.07^{b}

References

S1. Rajaram, R.; Chaitany, J. R.; Seong-Cheol, K.; Subramaniyan, R.; Tae, H. O.; Kuppusamy, M.; Yong, R. L. Encapsulation of tannic acid in polyvinylidene fluoride mediated electrospun nanofibers and its antibiofilm and antibacterial activities. J. Biomat. Sci. Pol. Ed. 2023, 34, 1911-1927.

S2. Sivakumar, A. S.; Sidong, K.; Inho, H. Cell-Cell Communication Between Fibroblast and 3T3-L1 Cells Under Co-culturing in Oxidative Stress Condition Induced by H_2O_2. Appl. Biochem. Biotech. 2016, 180, 668–681.

S3. Rajan, R.; Chandran, K.; Sivakumar, A.S.; Palaniappan, P.; Abhay Kumar, V.K.; Kwan Seob, S.; Chul-Gyu, S.; Soon-Il, Y. Anticancer activity of biologically synthesized silver and gold nanoparticles on mouse myoblast cancer cells and their toxicity against embryonic zebrafish. Mat. Sci. Eng. C. 2017, 73, 674-683.

S4. Chaitany, J. R.; Vinit, R.; Jin-Hyung, L.; Jintae, L. Antifungal activities of fluoroindoles against the postharvest pathogen Botrytis cinerea: In vitro and in silico approaches. Int. J. Food. Microbiol. 2022, 362, 109492.

S5. Chaitany, J. R.; Thirukumaran, P.; Rajesh, H.; Shakila, P. A.; Vinit, R.; Seong-Cheol, K. Synthesis of bio-based polybenzoxazine and its antibiofilm and anticorrosive activities. Materials, 2023, 16, 2249.

S6. Hamada El-Gendi, Ahmed Salama, Esmail M. El-Fakharany, Ahmed K. Saleh, Optimization of bacterial cellulose production from prickly pear peels and its ex situ impregnation with fruit byproducts for antimicrobial and strawberry packaging applications, Carbohydrate Polymers, 2023, 302, 120383.

S7. Allur Subramaniyan, S.; Chinzorio, O.; Soo-Hyun, C.; Jieun, Y.; Inho, H. Antiapoptotic effect of a novel synthetic peptide from bovine muscle and MPG peptide on H_2O_2-induced C2C12 cells, In Vitro Cellular and Developmental Biology – Animal. 2014, 50, 630–639.
